# Antifungal Properties of *Fucus vesiculosus* L. Supercritical Fluid Extract Against *Fusarium culmorum* and *Fusarium oxysporum*

**DOI:** 10.3390/molecules24193518

**Published:** 2019-09-28

**Authors:** Katarzyna Tyśkiewicz, Renata Tyśkiewicz, Marcin Konkol, Edward Rój, Jolanta Jaroszuk-Ściseł, Krystyna Skalicka-Woźniak

**Affiliations:** 1 Supercritical Extraction Department, ŁUKASIEWICZ Research Network—New Chemical Syntheses Institute, Tysiąclecia Państwa Polskiego Ave. 13a, 24-110 Puławy, Poland; marcin.konkol@ins.pulawy.pl (M.K.); edward.roj@ins.pulawy.pl (E.R.); 2 Department of Environmental Microbiology, Maria Curie-Sklodowska University, Akademicka St. 19, 20-033 Lublin, Poland; jolanta.jaroszuk-scisel@poczta.umcs.lublin.pl; 3 Department of Pharmacognosy with Medicinal Plant Unit, Medical University of Lublin, Chodźki St. 1, 20-093 Lublin, Poland; kskalicka@pharmacognosy.org

**Keywords:** brown algae, fucosterol, macroalgae, *Fusarium*, phytopathogens, supercritical fluid extraction

## Abstract

In this study, potential antifungal properties of a brown alga *Fucus vesiculosus* were evaluated. The algal extract was obtained with the use of supercritical fluid extraction (scCO_2_) at a temperature of 50 °C under a pressure of 300 bar. The aqueous solution of the extract at the concentration of 0.05%, 0.2%, 0.5% and 1.0% was studied against pathogenic fungi on a liquid RB medium. This study is the first report on antifungal properties of the brown algae *F. vesiculosus* scCO_2_ extract against *Fusarium culmorum* and *Fusarium oxysporum* phytopathogens. The concentrations of the studied extract (0.5% and 1.0%) were demonstrated to have an ability to inhibit 100% growth of macroconidia within 144 h, as well as an ability to cause their total degradation. As a result of the study, the antifungal effect of fucosterol against *F.* *culmorum* was also indicated. The total macroconidia growth was inhibited by 1.0% fucosterol. Moreover, at lower concentrations (0.05–0.2%) of fucosterol, macroconidia were characterized by shorter length and structural degradation was observed. The mycelial growth of *Fusarium oxysporum* (Fo38) by 1% scCO_2_ *F. vesiculosus* extract was analyzed at the level of 48% after 168 h of incubation, whereas 100% extract was found to be effective in *F. culmorum* (CBS122) and *F. oxysporum* (Fo38) growth inhibition by 72% and 75%, respectively after 168 h of incubation.

## 1. Introduction

Crops are exposed to diseases caused by pests and microorganisms during growth but also, after harvest. These factors can result in a large reduction in the annual level of food production worldwide, which depending on the source, is estimated at 25%–50% [1,2]. One-third of these losses are caused by fungal diseases. Currently, more than 10,000 species of fungi that can cause mycosis of plants have been classified, the most serious of which may be representatives of the genera *Botrytis*, *Rhizopus*, *Alternaria*, *Penicillium*, *Aspergillus*, *Rhizoctonia* and *Fusarium* [3,4].

Polyphagous fungi of the *Fusarium* genus, especially those belonging to the species of *Fusarium culmorum* and *Fusarium oxysporum* [5,6], are particularly dangerous for plants and human health. They are responsible for various types of fusarioses, including fusary wilting, snow mold, whitening of ears in field crops and greenhouse plants’ monocots (mainly *Poaceae*) and dicotyledons (including *Solanaceae*) [7]. These fungi synthesize toxins from the group of sesquiterpenes, such as deoxynivalenol (vomitoxin), nivalenol and zearalenone, which cause a variety of multiorgan diseases and also have a carcinogenic effect on humans during an intake of infected plants [5,8,9,10].

Phytopathogens infecting plants in one growing season may survive in the soil in the form of various propagules (both hyphae and spores) and then again may infect plants at various stages of their development. Phytopathogen propagules can be transported over long distances through the air as a component of bioaerosols [11].

Nowadays, effective and environmentally safe plant protection products against phytopathogenic fungi are sought [12], additionally plant protection products must, therefore, inhibit various forms of propagation [13]. Although synthetic chemicals are well known to have a fundamental role in the suppression of plant diseases and maintaining high crop yields, they have a harmful effect on humans and environmental integrity. The persistence of chemical pesticides is of major environmental concerns, thus natural products are considered to be less harmful to the environment due to their higher biodegradability and influential biocidal activities at lower doses [14,15]. Marine macroalgae are considered as an excellent source of bioactive compounds that have a broad range of biological activities, including antibacterial, antifungal, and antiviral properties, but are also used in agriculture as plant stimulators, as well as soil biofertilizer to enhance crop productivity [15]. It has been proven that algal extracts stimulate seed germination and growth, as well as the yields of various crops [16,17].

Preparations based on a variety of macerates and extracts from plant-like protista (algae) and blue-green algae exhibit a wide spectrum of activity against different classes of plant phytogenes and also inhibit germination of conidia (mycostatic activity), as well as vegetative hyphal growth and hyphae degradation with the use of lytic enzymes [18,19].

It has been shown that the antibacterial and antifungal properties of marine macroalgae are attributed to different groups of bioactive fatty acids, as well as phenolics [20,21]. Crude and purified algal preparations are able to induce the plant defense mechanism, reduce the incidence of the grey mold or different roots infections, but also, marine macroalgal extract-treated plants have been shown to increase the over-expression of some specific genes for defense signaling pathways [22].

There is already some evidence concerning the influence of different microalgae preparations on *Fusarium* fungi. In vivo utilization of *Spatoglossum variabile*, *Polycladia indica*, and *Melanothamnus afaqhusainii* have significant suppressive effects against the root-rotting fungi *Fusarium solani,* while in vivo application of *Padina gymnospora*, *Sargassum latifolium*, and *Hydroclathrus clathratus* powders, as soil amendments, decreased the percentage of root-rotting disease caused by *Fusarium solani* in *Solanum melongena* L. (eggplant) [20,23].

*Fucus vesiculosus* L. (Fucaceae), which belongs to the group of brown algae is a rich source of polysaccharides (fucoidans [24], laminarin [25]), and fucoxanthin [26], as well as valuable substances such as mannitol sugar alcohol [27] and sugar polymers [27]. These substances have harmful effects on phytopathogens but positive effects on plants and animals, and the electoral activity of these polymers and their oligosaccharide derivatives. Some preparations of different Fucus representatives have been applied previously. Methanolic extracts of *Fucus serratus* have been applied for large-scale production of biofertilizers due to their high content of betaines, an organic osmolytic compound that can potentially play a crucial role in effective protection against extreme environmental conditions [28]. Furthermore, in a greenhouse experiment, spray application of aqueous extracts from *Fucus spiralis* significantly reduced crown gall diseases caused by the bacterial pathogen *Agrobacterium tumefaciens* in tomato plants [19]. As for the *Fucus vesiculosus* extracts, the most studied extracts with antifungal properties are those obtained by polar solvent extractions [29], thus it would be interesting to check non-polar fraction activity obtained with the use of supercritical fluid extraction (SFE), as such studies have not been performed on *Fucus vesiculosus* yet. The literature data lack information on the influence of algal scCO_2_ extracts on fungi conidia.

The use of modern supercritical extraction methods with carbon dioxide (scCO_2_ SFE) guarantees the extraction of biologically active compounds without losing their properties and complete neutrality for the environment, plants and animals [30,31]. Supercritical fluid extracts of algae are mixtures of a number of active substances [30,32]. The SFE technique allows the extraction of various groups of compounds, including polyphenols [33], lipids (fatty acids, triacylglycerols) [34], micro- and macroelements [30], vitamins [35], plant growth hormones [30] and other compounds [36].

The aim of this study was to evaluate the ability of the scCO_2_ *Fucus vesiculosus* extract to inhibit the germination of macroconidia and limit the mycelial growth of *Fusarium* phytopathogens. In addition, the activity of the extract towards total degradation and lysis of macroconidia was also investigated. Moreover, the influence of fucosterol against the *F. culmorum* strain was studied.

## 2. Materials and Methods

### 2.1. Materials

All reagents for the RB (Reyes and Byrde medium) [37] medium preparation (KH_2_PO_4_, MgSO_4_·7H_2_O, KCl, (NH_4_)_2_SO_4_, glucose, Na_2_B_4_O_7_·10H_2_O, FeSO_4_·7H_2_O, MnSO_4_·5H_2_O, (NH_4_)_6_Mo_7_O_24_·4H_2_O, ZnSO_4_·7H_2_O) were purchased from POCH (Gliwice, Poland), apart from CuSO_4_·5H_2_O and streptomycin, which were purchased from REACHIM and Polfa (Lublin, Poland), respectively. Potato dextrose agar (PDA) was purchased from BIOMAXIMA (Lublin, Poland).

Experiments were carried out on two fungi strains of *Fusarium culmorum* (FcCBS122 and DEM Fc37), as well as two *Fusarium oxysporum* (FoCBS129 and DEM Fo38).

Both FcCBS122 and FoCBS129 standards, isolated from the tissues of cereal plants, were delivered by Centraalbureau voor Schimmelcultures Collections (CBS) P.O. Box 85167, NL-3508 AD Utrecht, The Netherlands. The pathogenic *F. culmorum* strain DEM Fc37 (CBS 120103, NCBI accession number for nucleotide sequence DQ450878) and *F. oxysporum* strain DEM Fo38 (CBS 120104, NCBI accession number for nucleotide sequence DQ450879) were isolated from fusariosis-infected wheat tissues and deposited at the Department of Environmental Microbiology’s Fungal Collection (DEM), Maria Curie-Skłodowska University (Lublin, Poland) [38]. The isolates were stored on Martin agar slants at 4 °C, according to the conditions provided by Jaroszuk-Ściseł et al. [39].

As for the extraction material, commercially available dried *Fucus vesiculosus* (NatVita Company, Mirków, Poland) was milled and subjected to supercritical fluid extraction (SFE). Fucosterol standard was purchased from Sigma-Aldrich (Poznań, Poland).

### 2.2. Preparation of Fucus vesiculosus Extract

The extraction was performed with a supercritical fluid extraction (SFE) system on a laboratory-scale installation with a working temperature of up to 80 °C and pressure of up to 450 bar. The system was equipped with a 1 L extractor. Dried and milled brown algae (*Fucus vesiculosus*) (200 g) were extracted with carbon dioxide. The temperature and pressure were set at 50 °C and 300 bar, respectively. The method was used according to the one described in our previous study [40]. The extraction resulted in 2.83 wt% extraction efficiency.

### 2.3. Growth Rate of Fusarium Strains on the Medium with Extract

To determine the effect of the scCO_2_ *F. vesiculosus* extract on the growth and development of phytopathogenic *Fusarium* strains, mycelia discs (0.8 cm) from initial cultures (grown on PDA medium for 7 days at 20 °C) were transferred to PDA medium with the addition of 100% extract (30 mg was spread on the agar surface using cell spreader) and 1% extract (1 mL of aqueous suspension of the extract was spread on the agar surface using cell spreader) in petri dishes (total diameter of 9.0 cm). At the same time, control versions (PDA medium without extract) were prepared for each studied strain. The petri dishes were incubated at 20 °C for 72 h, 96 h, 120 h and 168 h. After 72 h, 96 h, 120 h and 168 h of incubation, the diameters of the mycelium were measured and the R factor of the growth rate was calculated from the formula and was presented as mm^2^ mycelium/day (1). The IC_50_ (half-maximal inhibitory concentration) of *Fucus vesiculosus* scCO_2_ extract and fucosterol was also provided. The percentage inhibition of mycelium growth of *Fusarium* strains in a comparison with the control was determined.

(1) R=[(D2)2− (d2)2] · πT

where: R—growth rate factor; D—diameter of the mycelium (mm); d—mycelia discs (8.0 mm); π—3,14; T—incubation time (day).

### 2.4. Preparation of RB Liquid Medium

A liquid RB medium was prepared by mixing the following components KH_2_PO_4_ (1.0 g/L), MgSO_4_·7H_2_O (0.5 g/L), KCl (0.5 g/L), (NH_4_)_2_SO_4_ (0.5 g/L), glucose (10.0 g/L) and then dissolving in 1 L of distilled water. The medium was then sterilized at a temperature of 121 °C and pressure of 0.75 atm for 30 min. After autoclaving, the medium was supplemented with a separately prepared sterile mixture of microelements, including Na_2_B_4_O_7_·10H_2_O (100.0 mg/100 mL), CuSO_4_·5H_2_O (10.0 mg/100 mL), FeSO_4_·7H_2_O (50.0 mg/100 mL), MnSO_4_·5H_2_O (10.0 mg/100 mL), (NH_4_)_6_Mo_7_O_24_·4H_2_O (10.0 mg/100 mL) and ZnSO_4_·7H_2_O (70.0 mg/mL). The streptomycin (antibiotic; 30.0 mg/L) was added to the medium upon cooling (less than 60 °C) in order to avoid its decomposition.

### 2.5. Preparation of Fusarium Strains Macroconidia

Macroconidia of studied *F. culmorum* (FcCBS122 and DEM Fc37) and *F. oxysporum* (FoCBS129 and DEM Fo38) strains used for the preparation of inocula were obtained from a culture grown on a liquid RB medium with 1.0% glucose as a carbon source. The isolates were cultivated in darkness at 20 °C and 60% relative humidity in an Innova 4900 growth chamber (New Brunswick Scientific, Edison, NJ, USA) at 120 rpm for 7 days. Then, the fungal cultures were filtered through 5 layers of a sterile cotton gauze. The macroconidia were obtained by centrifuging the supernatants (10,000 g for 15 min). The supernatant was then collected and the resulting conidia pellet was resuspended in sterile distilled water. The density of the macroconidia suspension was determined in a hemocytometer using a light microscope (Olympus BX53 Upright Microscope) and then diluted with sterile distilled water to a desired concentration [38].

### 2.6. Preparation of Final Samples

The extract of *F. vesiculosus* was prepared with a concentration of 2.0% (*w*/*v*) by suspending 400 mg of extract in distilled water (20 mL). Correspondingly, *F. vesiculosus* extract at concentrations of 0.05%, 0.2%, 0.5% and 1.0% was also prepared. A macroconidia suspension (500 μL) of the *F. culmorum* DEM Fc37 strain (at a concentration of 2.0 × 10^6^ per mL) was transferred to four separate sterile Eppendorf tubes, followed by centrifugation (10,000 g for 15 min) on a centrifuge. The supernatant was then collected and the macroconidia pellet was supplemented with 500 μL of the extract at concentrations of 0.05%, 0.2%, 0.5% and 1.0%. Similarly, the samples of *F. culmorum* CBS122, *F. oxysporum* DEM Fo38, *F. oxysporum* CBS129 were prepared. In the experimental version with the RB medium, a macroconidia suspension (500 μL) of the *F. culmorum* DEM Fc37 strain (at a concentration of 2.0 × 10^6^ per mL) was also transferred to four separate sterile Eppendorf tubes, followed by centrifugation (10,000 g for 15 min) on a centrifuge. The supernatant was then collected and the macroconidia pellet was supplemented with 250 μL of 2.0% extract and 250 µL of the RB medium to obtain a concentration of 1.0%. Similarly, the samples of 0.5%, 0.2% and 0.05% extract was obtained by mixing 125 µL of 2.0% extract + 375 µL of the RB medium, 50 µL of 2.0% extract + 450 µL of the RB medium and 12.5 µL of 2.0% extract + 487.5 µL of the RB medium, respectively.

### 2.7. Preparation of Fucosterol Standard

**Figure 1:**
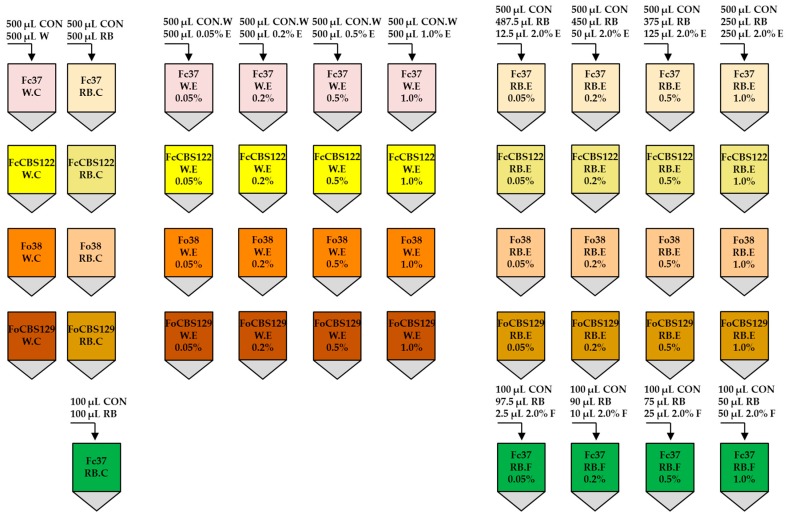
The diagram of studied samples (W—water; RB—medium; W.C—water control; RB.C—RB control; E—extract; F—fucosterol; CON—conidia; CON.W—conidia suspended in water).

The fucosterol standard was prepared with a concentration of 2.0% (*w*/*v*) by suspending 2.0 mg extract in distilled water (98 μL). A macroconidia suspension (100 μL) of the *F. culmorum* DEM Fc37 strain (at a concentration of 2.0 × 10^6^ per mL) was transferred to four separate sterile Eppendorf tubes, followed by centrifugation (10,000 g for 15 min) on a centrifuge. The supernatant was then collected and the macroconidia pellet was supplemented with 50 μL of the RB medium and 50 μL of 2.0% fucosterol to obtain a concentration of 1.0%. Similarly, samples of 0.5%, 0.2% and 0.05% fucosterol were obtained by mixing 75 μL RB medium + 25 μL 2.0% fucosterol, 90 μL RB medium + 10 μL 2.0% fucosterol and 97.5 μL RB medium + 2.5 μL 2.0% fucosterol, respectively. Figure 1 presents the schema of studied samples. The samples names are used throughout the text.

### 2.8. Evaluation of Macroconidia Germination Capacity of Fusarium spp.

After 24 h, 48 h, 72 h, 96 h, 120 h and 144 h of incubation in darkness at 20 °C and 60% relative humidity in an Innova 4900 growth chamber (New Brunswick Scientific, Edison, NJ, USA) at 120 rpm, 30 μL of each sample was applied on a sterile slide, observed and photographed using an Olympus BX53 Upright Microscope equipped with an Olympus XC30 camera. The presented results are the percentage of germinated macroconidia and the average length of hyphae of 300 conidia from 3 independent experiments/replicates (100 spores per one repetition) observed in 10 different microscopic fields. The obtained results were calculated in relation to an appropriate control (water, RB).

### 2.9. Statistical Analysis

The statistical analyses were conducted using Statistica 12.5 (StatSoft Inc., Kraków, Poland). All assays were performed in three independent experiments and the data were expressed as means ± SD calculated from these experiments. The data were subjected to one-way analysis of variance (ANOVA) followed by a Tukey’s post hoc test, with the significance evaluated at *p* < 0.05.

## 3. Results and Discussion

### 3.1. The Effect of F. vesiculosus Extract on the Mycelial Growth of Fusarium Strains

**Figure 2:**
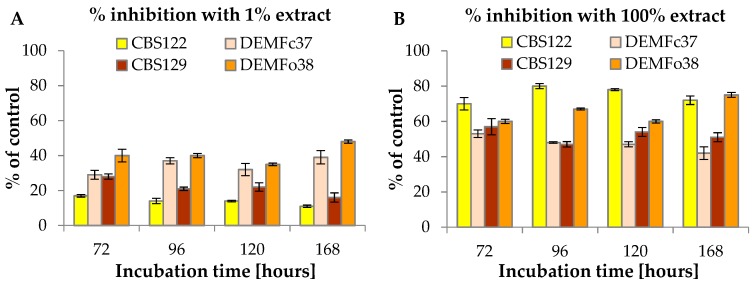
The percentage inhibition of mycelial growth of *Fusarium culmorum* (CBS122, Fc37) and *Fusarium oxysporum* (CBS129, Fo38) strains on PDA medium with addition of (**A**) 1% and (**B**) 100% *F. vesiculosus* extract, after 72 h, 96 h, 120 h and 168 h of incubation in a comparison with the control.

The scCO_2_ *F. vesiculosus* extract significantly limited the mycelial growth of phytopathogenic *Fusarium* strains. The value of R factor of the CBS122, Fc37, CBS129 and Fo38 strains was lower by about 1.2, 1.5, 1.4 and almost 2 times lower on the medium with the addition of 1% extract, as well as 5-, 2-, 2.3- and 4-times lower on the medium with the addition of 100% extract in a comparison with the control, respectively (Table 1). Figure 2 presents the effect of 1% and 100% *F. vesiculosus* extract on the inhibition of mycelial growth of *F. culmorum* and *F. oxysporum*. In the case of 1% *F. vesiculosus*, the inhibition of mycelial growth of *F. culmorum* was at the level of 17% (72 h) to 11% (168 h) and 29% (72 h) to 39% (168 h), respectively, for CBS122 and Fc37. The inhibition of CBS129 and Fo38 was at the level of 28%–16% and 40%–48% from 72 h to 168 h of incubation, respectively. The mycelial growth of *F. culmorum* was strongly and significantly inhibited by 100% *F. vesiculosus* extract as compared to 1% extract. For instance, the inhibition of *F. culmorum* and *F. oxysporum* after 72 h was 70%, 53%, 57%, and 60%, respectively, for CBS122, Fc37, CBS129 and Fo38. The highest inhibition of CBS122 (80%) was observed after 96 h of incubation treated with 100% extract, whereas Fc37 was inhibited at the highest rate (53%) after 72 h of incubation. As for *F. oxysporum*, the treatment with 100% extract resulted in the highest inhibition of CBS129 (57%) and Fo38 (75%) after 72 h and 168 h of incubation, respectively.

**Figure 3:**
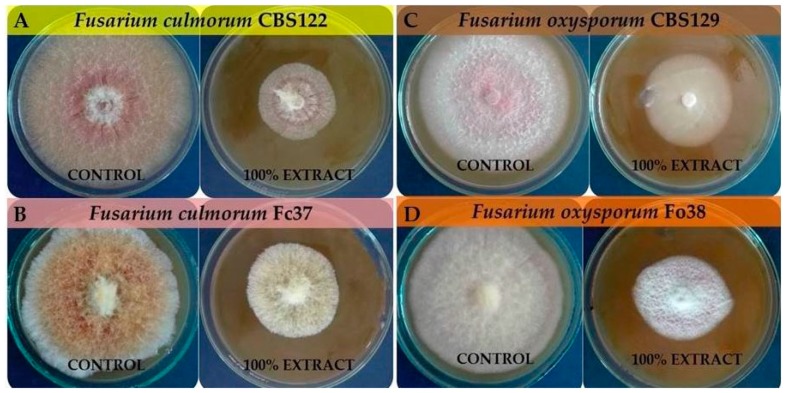
The effect of 100% *F. vesiculosus* extract on *F. culmorum* (CBS122 (**A**), Fc37 (**B**)) and *F. oxysporum* (CBS129 (**C**), Fo38 (**D**)) on PDA medium after 168 h of incubation.

Nowadays, the most common method for plant protection against phytopathogenic fungi is the use of chemical fungicides. These products are characterized by a high effectiveness, however, concerns regarding the safety and health of food mean that alternative methods of combating phytopathogens are being sought [41]. In order to limit the negative effects of the use of non-biological plant control agents, studies are being conducted to develop environmentally friendly crop technologies based on the use of soil microorganisms or their metabolites and natural products (plants extracts) in accordance with the assumption of integrated pest management (IPM) and organic farming [42,43]. Manni et al. [44] studied the influence of five chemical fungicides, such as Prodazin (Carbendazim), Dithane (mancozeb), Alliette Express (Fosetyl-Al), Tachigazol (Hymexazol) and Beltanol (Chinosol) in the concentration range of 10, 25, 50 and 100 ppm against *Fusarium oxysporum*. The best results were observed for Tachigazol and Beltanol, with the highest *F. oxysporum* inhibition ability among all the tested fungicides being that of Beltanol. After 144 h of incubation, *F. oxysporum* was inhibited by over 90% and 70%, respectively, by Beltanol (100 ppm) and Tachigazol (100 ppm). The least effective was Alliette Express (Fosetyl-Al) with *F. culmorum* inhibition by 7.03% (10 ppm) and 0.61% (100 ppm). In another study, the increase of *Fusarium* growth inhibition was noticed with the increase of the concentrations of fungicides, such as propiconazole (250 g/L), metconazole (60 g/L) and tebuconazole (250 g/L) [45]. In the triazole group, metconazole showed the strongest inhibitory effect on the growth of all tested fungi. The growth of *Fusarium culmorum* and *Fusarium oxysporum* was inhibited by 100% and 70%–100%, respectively. The results provided in this study, indicate that *F. vesiculosus* scCO_2_ extract may be used as a potential additive to biological plant protection products as the extract was characterized by a high inhibitory effect on *Fusarium culmorum* and *Fusarium oxysporum*, similar to the effect caused by the chemical fungicides. Moreover, the extracts obtained with the use of supercritical carbon dioxide are free of solvents, as carbon dioxide is removed by depressurization [30]. Figure 3 presents the effect of 100% *F. vesiculosus* extract on *F. culmorum* (CBS122, Fc37) and *F. oxysporum* (CBS129, Fo38) on PDA medium after 168 h of incubation.

### 3.2. The Effect of F. vesiculosus Extract on the Fusarium Macroconidia

**Figure 4:**
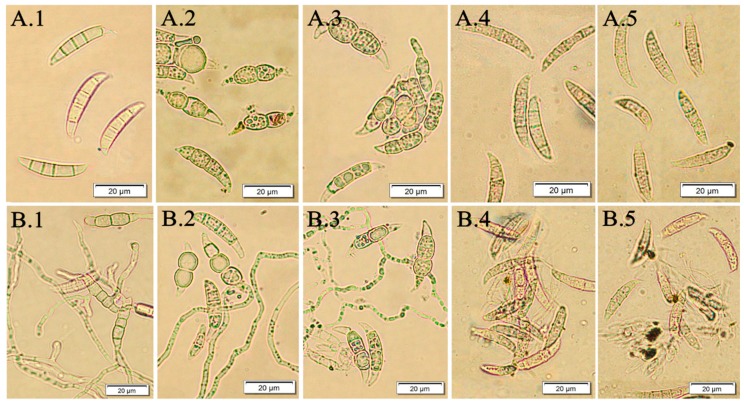
*F. culmorum* DEMFc37 treated with *F. vesiculosus* water extract (W.E) after 120 h of incubation; (**A**) non-germinated; (**B**) germinated macroconidia with hyphae of different length; (1) water control (W.C); (2) 0.05%; (3) 0.2%; (4) 0.5 %; (5) 1.0%.

The present investigations demonstrated that the *F. vesiculosus* scCO_2_ extract tested had a high in vitro antifungal activity against *F. culmorum* and *F. oxysporum* macroconidia. The susceptibility of studied pathogens towards the brown algae extract was reflected in a trend received from two quantitative bioassays (fungal macroconidia germination and hyphae length). The best efficacy was when pathogens were treated with the *F. vesiculosus* extract at concentrations of 0.5% and 1.0%. The same effect was observed for all tested pathogens (*F. culmorum* Fc37, *F. culmorum* CBS122, *F. oxysporum* Fo38, *F. oxysporum* CBS129). Figure 4 presents the microscopic view of *F. culmorum* DEMFc37 macroconidia treated with the *F. vesiculosus* extract at the concentration of 0.05%‒1.0% after 120 h of incubation.

The antifungal properties exhibited by marine algae may be attributed to the presence of biologically bioactive compounds, including polysaccharides and derived oligosaccharides, lipids, fatty acids, sterols, phenolic compounds, pigments, lectins, alkaloids, and terpenes, as well as halogenated compounds (furanones, bromoditerpenes, bromophenols) [46]. Antifungal activities of particular bioactive compounds have been reported previously [47,48,49]. In the case of brown algae, in particular, fucoidans are the main group belonging to polysaccharides [50]. In a review on *Fucus* spp. by Catarino et al. [25], the content of fucoidans, alginic acid and laminaran in *F. vesiculosus* may be in the range of 3.4%–25.7%, 8.4%–58.5% and 0.6–7.0 dry weight, respectively. According to our previous study [40], *Fucus vesiculosus* scCO_2_ extract contains fucosterol in the amount of 8.05 wt%. The microwave-processed extract of *F. vesiculosus* was studied in terms of the production of fucoidanase enzymes by a solid-state fermentation with two fungal strains, such as *Aspergillus niger* and *Mucor* sp. [51]. However, according to Alexeeva et al. [52], fucoidans are described as low-activity compounds.

**Figure 5:**
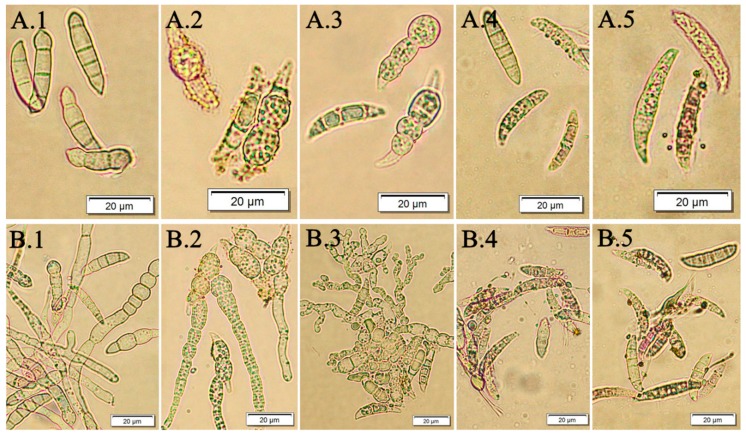
*F. culmorum* DEMFc37 treated with *F. vesiculosus* RB extract (RB.E) after 120 h incubation, (**A**) non-germinated; (**B**) germinated macroconidia with hyphae of different length; (1) RB control (RB.C); (2) 0.05%; (3) 0.2%; (4) 0.5%; (5) 1.0%.

Two experiments were carried out in order to examine the behavior of fungi in two environments. The water control (W.C) for the first experiment contained distilled water with particular *Fusarium* macroconidia, whereas for the second one, the control (RB.E) constituted macroconidia on a liquid RB medium. The aim of the second bioassay was to evaluate the influence of the *F. vesiculosus* extract on *Fusarium* conidia when the conditions were favourable for their growth as they could have taken up nutrients from the RB medium. The visual effect is shown in Figure 5. The obtained results of the percentage of germinated macroconidia, as well as the percentage of the average length of hyphae, showed a consistent fungistatic and antifungal effect. The extract concentrations of 0.5% and 1.0% were more effective than that of 0.05% and 0.2%. After 10 days of the experiment, *F. vesiculosus* scCO_2_ extract at a concentration of 1.0% still showed a strong inhibitory effect on all studied *Fusarium* conidia.

**Figure 6:**
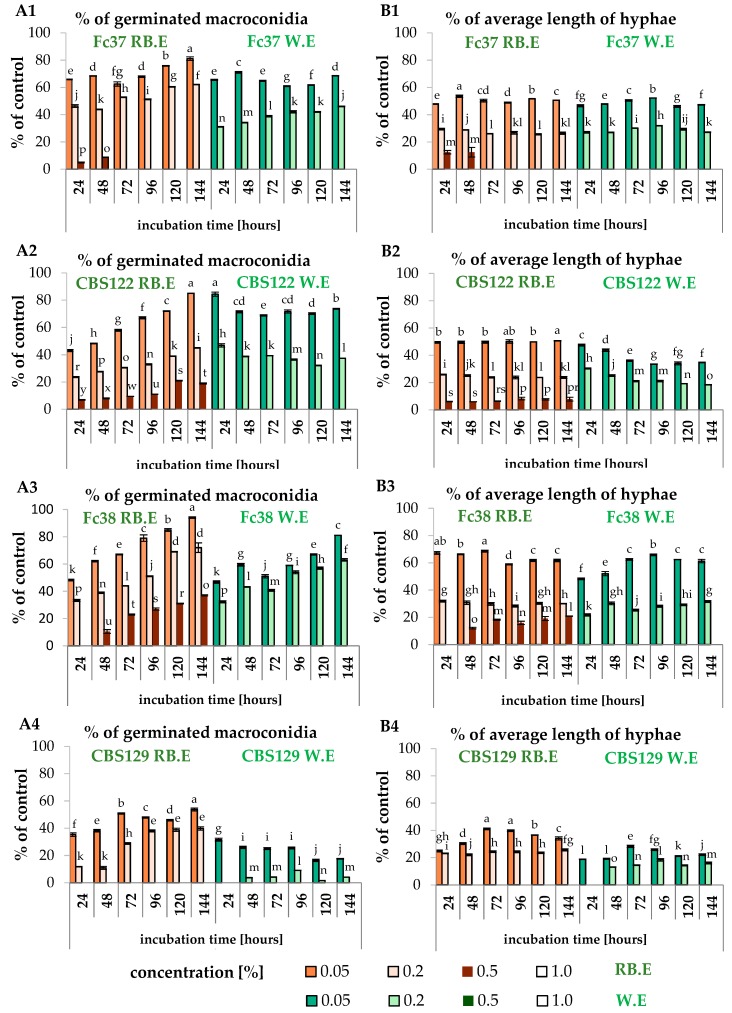
The percentage of germinated macroconidia (**A**) and the percentage of average hyphae length (**B**) of *F. culmorum* (1 for *F. culmorum* Fc37; 2 for *F. culmorum* FcCBS122) and *F. oxysporum* (3 for *F. oxysporum* Fo38; 4 for *F. oxysporum* FoCBS129). Bars represented standard deviations (SD). Values followed by different letters are significantly different (one-way analysis of variance (ANOVA) followed by a Tukey’s post hoc test with the significance evaluated at *p* < 0.05).

In the case of *F. culmorum* Fc37, the germination of macroconidia was strongly inhibited when 0.5% and 1.0% extracts were applied, which also corresponded to no increase of average length of hyphae in an experiment with the extract and conidia (Fc37 W.E). It was observed that 0.5% extract in Fc37 RB.E samples was effective after 48 h of incubation. It may be assumed that the spores of *F. culmorum* Fc37 were able to feed themselves with RB-medium nutrients within 48 h. Similarly to Fc37, the spores of *F. culmorum* CBS122 were proven to be inhibited by the addition of 0.5% and 1.0% extract in FcCBS122 W.E samples, whereas FcCBS122 RB.E samples were characterized by the percentage of germinated spores in the range of 7%–21% (24–144 h of incubation), as well as the average percentage of hyphae length at the level of 6%–8% (24–144 h of incubation) (Figure 6(A1, B1, A2, B2)).

The growth of *F. oxysporum* Fo38 was fully inhibited by the *F. vesiculosus* scCO_2_ extract at the concentration of 0.5% and 1.0%. The samples of Fo38 W.E were characterized by no increase of both germinated macroconidia and mean length of hyphae upon treatment with the extract, according to an appropriate control (W.C, RB.C). Similarly to Fo38 W.E, the same effect of the 0.5% and 1.0% extracts was observed for the FoCBS129 W.E and FoCBS129 RB.E samples. In the case of Fo38 RB.E, the most effective concentration of *F. vesiculosus* extract in the inhibition of macroconidia growth was 1.0%. The treatment of Fo38 RB.E with 0.5% extract resulted in an increase by 0%–37% of germinated spores and by 0%–21% of mean length of hyphae within 0–144 h of incubation (Figure 6(A3, B3, A4, B4)). 

**Figure 7:**
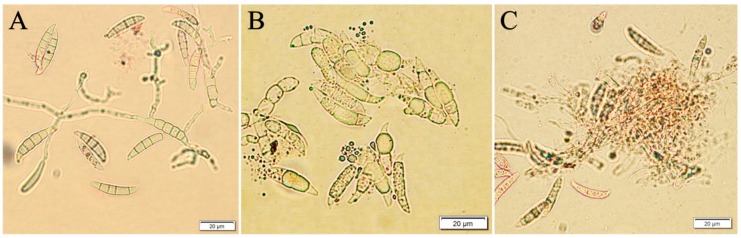
Degradation of *F. culmorum* Fc37 macroconidia in W.E: (**B**) on the example of Fc37 W.E 0.2%; (**C**) Fc37 W.E 1.0% in a comparison to (**A**) water control (W.C), 96 h of incubation.

Like other *Fusarium* species, *F. culmorum* and *F. oxysporum* are known for their ability to survive in the soil, even for several years, especially on post-harvest residues, due to inoculum production [53,54]. Moreover, both fungi species (*F. culmorum* and *F. oxysporum*) are characterized by a low degradation risk as they have an ability to survive in adverse conditions by using defense mechanisms. Furthermore, *Fusarium* species are responsible for causing fusariosis, which is one of the most serious infections of plants, especially cereal plants [55]. According to Lionakis and Kontoyiannis [56], biological plant protection products are effective to some extent due to an innate resistance of pathogens to such preparations. On the other hand, more drastic methods include the use of chemical protection products that have a negative impact on the environment [57]. The promising agents for combating these pathogens may be algal-based preparations. Apart from its properties to inhibit *F. culmorum* and *F. oxysporum* macroconidia growth, the *F. vesiculosus* scCO_2_ extract was also found to be effective in terms of total degradation and lysis (Figure 7) of Fc37, FcCBS122, Fo38 and FoCBS129 macroconidia. Our study is the first to prove the degradation and lysis of macroconidia caused by the *F. vesiculosus* scCO_2_ extract. The lysis was performed using extract even at the concentration of 0.2%, as presented in Figure 7.

### 3.3. The Effect of Fucosterol on the Fusarium Macroconidia

**Figure 8:**
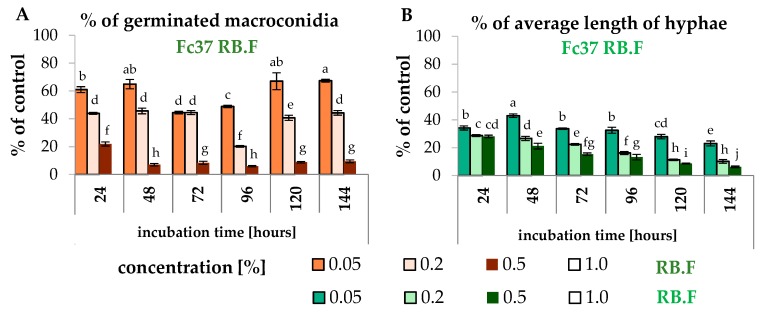
The percentage of germinated macroconidia and the percentage of the average hyphae length of *F. culmorum* treated with fucosterol, the percentage of germinated macroconidia (**A**) and the percentage of average hyphae length (**B**) Bars represented standard deviations (SD). Values followed by different letters are significantly different (one-way analysis of variance (ANOVA) followed by a Tukey’s post hoc test with the significance evaluated at *p* < 0.05).

The experiment to determine the influence of the fucosterol standard on *F. culmorum* Fc37 on an RB medium was carried out in order to evaluate the antifungal properties of one of *F. vesiculosus* scCO_2_ extract components. According to our previous study, the content of fucosterol in the extract was 8.06 wt% [40]. On the basis of the results from this study, it may be concluded that fucosterol significantly affected the growth of *F. culmorum* strains. The germination of *F. culmorum* Fc37 macroconidia was fully inhibited by fucosterol at the concentration of 1.0% in the Fc37 RB.F sample. In the case of the Fc37 RB.F sample with 0.5% fucosterol, the percentage of germinated macroconidia was in the range of 5.95%–21.95%, whereas the percentage of hyphae length was in the range of 6.25%–28.12%. After 24 h of incubation of Fc37 RB.F 0.5% sample, the highest germination of macroconidia (21.95%) was observed, which was further decreased over two times (9.47%) after 144 h of incubation (Figure 8A,B).

**Figure 9:**
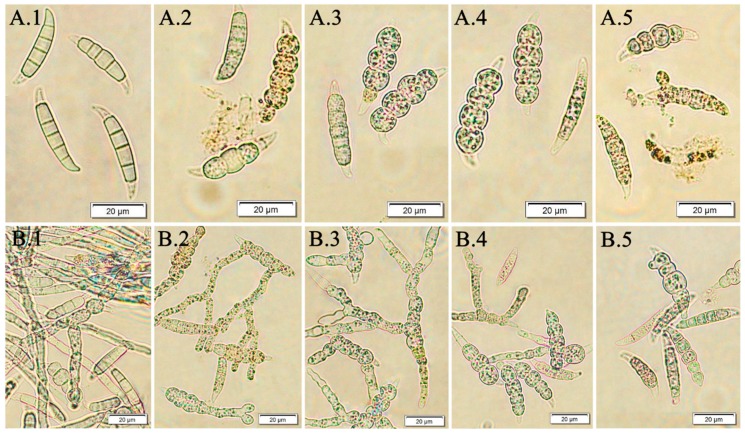
*F. culmorum* DEMFc37 non-germinated (**A**) and germinated (**B**) macroconidia treated with (1) 0.05%, (2) 0.2 %, (3) 0.5% and (4) 1.0% fucosterol after 120 h of incubation.

Similarly to the *F. vesiculosus* scCO_2_ extract, fucosterol at a concentration of 1.0% caused total degradation, as well as lysis of *F. culmorum* Fc37 conidia, in the Fc37 RB.F sample. In the case of lower concentrations of fucosterol (0.05%–0.5%), the deformation of Fc37 macroconidia was observed in all samples (Fc37 RB.F 0.05%, Fc37 RB.F0.2%, Fc37 RB.F0.5%). The percentage of germinated macroconidia treated with fucosterol in Fc37 RB.F 0.2% and Fc37 RB.F 0.05% samples was 44.21% and 67.37%, respectively. Even though the growth of macroconidia was partially inhibited by fucosterol, the conidia were observed to grow. However, they were much shorter and deformed in a comparison with the control (Figure 9). According to Newman et al. [58], sterols (cholesterol, ergosterol) may take part in lipid bilayer modifications in terms of both structural and thermodynamic properties, causing a liquid-ordered (lo) phase separation. Moreover, sterols are said to react with membrane components and enzymes, enabling their activation or causing their inactivation [59]. The literature reports the antifungal properties of β-sitosterol, campesterol and stigmasterol form the extract of *Dispacus asper* against phytopathogenic fungi, such as *Botrytis cinerea*, *Puccinia recondita* and *Rhizoctonia solani* [60]. The fungal pathogens are suggested to survive the effect of active compounds (for instance, azole) due to the ability to decrease the accumulation of these compounds by different defense mechanisms [61]. Both Papadopoulou et al. [62] and Upadhyay et al. [63], indicated the potential antifungal effect of saponins. 

Table 2 presents the half-maximal inhibitory concentration for *Fucus vesiculosus* scCO_2_ extract and fucosterol. The IC_50_ values for the extract in the RB.E experiment were in the range of 0.02%–0.25%, with the highest concentration of the extract against CBS129 and DEMFo38. In the case of W.E experiment, the highest IC50 value (1.82%) was obtained against CBS129. The IC_50_ for fucosterol against DEMFc37 was calculated at the level of 0.06%.

## 4. Conclusions

Due to its strong inhibition properties against pathogenic fungi, supercritical carbon dioxide extract of *F. vesiculosus* may be used as a potential component of a biological plant protection product, especially against phytopathogenic fungi of *Fusarium* spp. These fungi are particularly dangerous for various crops in all geographical regions.

## Figures and Tables

**Table 1:** The growth rate of *Fusarium culmorum* (CBS122, Fc37) and *Fusarium oxysporum* (CBS129, Fo38). strains on potato dextrose agar (PDA) medium with addition of 1% and 100% *F. vesiculosus* extract after 72, 96, 120 and 168 h of incubation in a comparison with the control. The growth rate was presented as R factor (mm^2^ mycelium/day).

Incubation Time [h]	*Fusarium culmorum*					
	CBS122			Fc37		
	R Factor (mm^2^ mycelium/day)					
	Control	Extract 1%	Extract 100%	Control	Extract 1%	Extract 100%
**72**	322.37 ± 8.23	268.21 ± 12.54	98.65 ± 5.76	285.74 ± 12.45	203.32 ± 5.76	133.97 ± 9.54
**96**	625.06 ± 23.14	538.71 ± 9.54	120.11 ± 10.54	358.79 ± 11.56	227.85 ± 14.76	188.40 ± 4.87
**120**	873.08 ± 15.67	759.35 ± 19.94	193.42 ± 5.76	430.97 ± 9.76	293.90 ± 10.12	228.75 ± 12.04
**168**	901.18 ± 8.32	803.06 ± 15.04	251.20 ± 12.67	590.43 ± 19.43	357.18 ± 11.04	344.50 ± 13.76
	***Fusarium oxysporum***					
	**CBS129**			**Fo38**		
**72**	203.32 ± 3.98	146.80 ± 13.55	87.92 ± 6.87	146.80 ± 11.67	87.92 ± 3.67	58.88 ± 8.65
**96**	285.94 ± 12.56	227.85 ± 9.04	152.49 ± 12.11	221.03 ± 20.02	130.51 ± 9.54	73.99 ± 7.22
**120**	374.64 ± 10.54	293.90 ± 6.76	171.44 ± 13.54	280.25 ± 14.67	182.28 ± 11.05	113.04 ± 2.54
**168**	511.37 ± 10.32	437.92 ± 17.51	251.20 ± 10.54	396.54 ± 17.45	209.93 ± 9.94	100.59 ± 7.43

**Table 2:** The IC_50_ of *Fucus vesiculosus* scCO_2_ extract and fucosterol.

Strains	Mycelial Growth	Macroconidia Germination		
		*F. vesiculosus* scCO_2_ Extract		Fucosterol
		RB.E	W.E	RB.F
	IC_50_ [%]			
**CBS122**	59.63 ± 4.32	0.02 ± 0.00	0.13 ± 0.01	
**DEMFc37**	120.12 ± 4.75	0.19 ± 0.07	0.07 ± 0.00	0.06 ± 0.00
**CBS129**	93.21 ± 5.41	0.25 ± 0.09	1.82 ± 0.12	
**DEMFo38**	38.43 ± 6.22	0.25 ± 0.03	0.09 ± 0.00	
